# Reporter-Based Assays for High-Throughput Drug Screening against *Mycobacterium abscessus*

**DOI:** 10.3389/fmicb.2017.02204

**Published:** 2017-11-10

**Authors:** Rashmi Gupta, Mandy Netherton, Thomas F. Byrd, Kyle H. Rohde

**Affiliations:** ^1^Division of Immunity and Pathogenesis, Burnett School of Biomedical Sciences, College of Medicine, University of Central Florida, Orlando, FL, United States; ^2^Division of Infectious Diseases, Department of Internal Medicine, The University of New Mexico School of Medicine, Albuquerque, NM, United States

**Keywords:** non-tuberculous mycobacteria, drug discovery and development, drug discovery screening, luminescence, fluorescence, reporter genes

## Abstract

*Mycobacterium abscessus* is a non-tuberculous mycobacterium that causes pulmonary and non-pulmonary infections. *M. abscessus* is resistant to many chemotherapeutic agents and the current treatment options show poor clinical outcomes. Thus, there is a dire need to find new antimicrobials effective at killing *M. abscessus*. Screening drug libraries to identify potential antimicrobials has been impeded by the lack of validated HTS assays for *M. abscessus*. In this study, we developed two 384-well high-throughput screening assays using fluorescent and bioluminescent reporter strains of *M. abscessus* for drug discovery. Optimization of inoculum size, incubation time and the volume-per-well based on Z-factor and signal intensity yielded two complementary, robust tools for *M. abscessus* drug discovery with Z-factor > 0.8. The MIC of known drugs, amikacin and clarithromycin, as determined by bioluminescence was in agreement with the published MIC values. A proof-of-concept screen of 2,093 natural product-inspired compounds was conducted using the 384-well bioluminescent assay to identify novel scaffolds active against *M. abscessus.* Five active “hit” compounds identified in this pilot screen were confirmed and characterized by a CFU assay and MIC determination. Overall, we developed and validated a 384-well screen that offers simple, sensitive and fast screening of compounds for activity against this emerging pathogen. To our knowledge, this is the first reporter–based high-throughput screening study aimed at *M. abscessus* drug discovery.

## Introduction

*Mycobacterium abscessus* (*M. abscessus*) is a non-tuberculous mycobacterium that causes a wide range of human diseases such as skin and soft tissue infections, disseminated disease and chronic respiratory infections (Howard and Byrd, 2000; Sanguinetti et al., 2001). *M. abscessus* is also an important pathogen in cystic fibrosis (CF) patients where it colonizes and infects the lungs, further exacerbating the condition of the patients. Data suggests that, in the environment as well as during an infection, *M. abscessus* can exist as two distinct morphotypes, exhibiting either a rough (R) or smooth (S) colony phenotype, depending on the presence or absence of glycopeptidolipids in their cell walls (Byrd and Lyons, 1999; Howard et al., 2006; Nessar et al., 2011). The smooth variant forms biofilms while both rough and smooth types are known to infect macrophages (Brambilla et al., 2016). These two variants also differ in their virulence potential with the R phenotype showing more aggressive behavior in pulmonary infections (Byrd and Lyons, 1999; Catherinot et al., 2009). Irrespective of the phenotype, *M. abscessus* infections are difficult to treat owing to the inherent resistance of this pathogen to many chemotherapeutic agents. Moreover, the current treatment with amikacin and clarithromycin for *M. abscessus* infections has shown poor efficacy in the clinical settings (Jarand et al., 2011; Lyu et al., 2011), despite potent activity against the bacilli *in vitro*. This highlights the need to identify new antimicrobials effective against *M. abscessus*.

Despite the urgent need for improved therapeutics for *M. abscessus* infections, drug discovery efforts targeting *M. abscessus* have been few and far between. There have been efforts to repurpose tuberculosis (TB) drugs considering the phylogenetic relatedness between *Mycobacterium tuberculosis* and *M. abscessus*. However, the lack of activity of many anti-tubercular drugs against *M. abscessus*, including the front-line TB drugs rifampicin (Rif) and isoniazid (Inh) (Abraham, 2007; Otchere et al., 2017), has limited the success of this approach. In other studies, incremental improvements of existing drugs by scaffold modification or formulation as multidrug cocktails have yielded a few new *M. abscessus* active compound series (Nessar et al., 2012; Benwill and Wallace, 2014; Baranyai et al., 2015; Kang and Koh, 2016; Waters and Ratjen, 2016). There is often a notable lack of correlation between potent *in vitro* inhibition of *M. abscessus* and *in vivo* efficacy, revealing an ignorance of the *in vivo* physiology of *M. abscessus*. These failures argue for the need for novel scaffolds with specific potency for *M. abscessus*. The screening of drug libraries offers exploitation of large chemical space with greater likelihood of finding novel drugs against *M. abscessus* as opposed to repurposing of existing drugs. Even then, the investment in large-scale primary drug screening against *M. abscessus* has been conspicuously lacking. In fact, there are no reported high throughput drug screens (HTS) except for two small scale studies testing either FDA approved or known TB active compounds against *M. abscessus* (Chopra et al., 2011; Low et al., 2017). Moreover, most *M. abscessus* discovery studies including those referenced above have relied on the traditional method of evaluating growth by visual inspection and/or by measuring absorbance in microplates. Some studies employed a more sensitive resazurin dye-based microplate method for quantitative assessment of antimicrobial activity against non-tuberculous mycobacteria (Taneja and Tyagi, 2007; Li et al., 2013). This dye-based antimicrobial susceptibility method requires more time and an additional liquid handling step unlike reporter-based whole-cell assays.

Reporter-based assays are well suited for high-throughput drug discovery applications owing to the simplicity and sensitivity compared to the dye-based and absorbance-based assays. Fluorescent mCherry (Ollinger et al., 2013; Stanley et al., 2014; Sandhaus et al., 2016) and bioluminescent markers have been developed and used effectively with *M. tuberculosis, Mycobacterium avium, Mycobacterium intracellulare*, and *Mycobacterium bovis* to test drug susceptibility both *in vitro* and in macrophages (Cooksey et al., 1995; Arain et al., 1996; Shawar et al., 1997; Cho et al., 2007; Ballell et al., 2013; Grant et al., 2013; Wang et al., 2016; Dalton et al., 2017). Surprisingly, few efforts have utilized reporters to examine drug activity against the highly drug resistant *M. abscessus*. To our knowledge, there are no published reports of HTS drug assays employing reporter strains of *M. abscessus*. In this study we developed and optimized fluorescent and bioluminescent reporter-based whole-cell drug screening assays in a 384-well format with the intent of screening large libraries. We then validated the HTS platform by testing a subset of compounds from an Asinex library that contains natural product-like compounds where key structural features of known pharmacologically relevant natural products (e.g., alkaloids and other secondary metabolites) are incorporated into synthetically feasible medicinal chemistry scaffolds. These tools for HTS drug evaluation of chemical libraries are likely to accelerate *M. abscessus* discovery programs.

## Materials and Methods

### Compounds and Library

Antibiotics amikacin (AMK) and clarithromycin (CLR) were purchased from Sigma-Aldrich and stock solutions were prepared as per the manufacturer’s instructions. A library of 2,093 natural-product like scaffolds found in the 50,000 BioCore (BioDesign) compounds were purchased from Asinex^1^. This set of unique chemotypes were chosen as representative compounds from each cluster identified from the larger BioCore library using the cheminformatics software package Molsoft ICM Chemistry Pro^2^ and JKlustor (ChemAxon).

### Bacterial Strains and Plasmids

*Mycobacterium abscessus* strains were a kind gift from Dr. Thomas Byrd from the University of New Mexico (Howard et al., 2006; Greendyke and Byrd, 2008; Brown-Elliott et al., 2012). *M. abscessus* 390S (smooth), and 390R (rough) were grown at 37°C in Middlebrook 7H9 supplemented with 10% OADC. Antibiotics [50 μg/ml kanamycin (KAN) or 32 μg/ml amikacin (AMK)] were added for selection when appropriate. *M. abscessus* 390R strain was passaged 5X with a tuberculin needle (21G) to reduce clumps before carrying out experiments.

*Mycobacterium abscessus* strains 390S and 390R were transformed with plasmids, episomal pVVRG (Rodrigues Felix et al., 2017) and integrative pMV306hsp+LuxG13 (Andreu et al., 2010) by electroporation. The former plasmid expresses mCherry and GFP constitutively from smyc and hsp60 promoters, respectively while the latter plasmid carries the entire *LuxCDABE* operon. The bioluminescent reporter plasmid is a kind gift from Brian Robertson & Siouxsie Wiles (Addgene # 26159). Transformants were selected on 7H10 plates supplemented with OADC and 50 μg/ml KAN. The resulting strains are referred to as 390S-mCh/390R-mCh and 390S-*lux*/390R-*lux* throughout this manuscript. **Table 1** details the bacterial strains and plasmids used in this study.

**Table 1:** Bacterial strains and plasmids.

Name	Genotype/Phenotype	Reference
**Plasmids**		
pVVRG	Episomal plasmid, mCherry and GFP fluorescence, Kan^R^	Rodrigues Felix et al., 2017
pMV306hsp+LuxG13	integrative plasmid, *luxCDABE*, Kan^R^	Addgene #26159, Andreu et al., 2010
**Strains**		
390S	*M. abscessus* strain, smooth colony phenotype	Byrd and Lyons, 1999
390R	*M. abscessus* strain, rough colony phenotype	Byrd and Lyons, 1999
390S-mCh	390S strain harboring pVVRG, constitutively express mCherry and GFP	This study
390S-*lux*	390S strain expressing *luxCDABE*, bioluminescent	This study
390R-mCh	390R strain harboring pVVRG, constitutively express mCherry and GFP	This study
390R-*lux*	390R strain expressing *luxCDABE*, bioluminescent	This study

### Reporter Plasmid Stability Assay

The stability of the reporter plasmids was assessed by comparing the signal as well as cell viability (by CFU) of *M. abscessus* strains, 390S-*lux* and 390S-mCh, when grown in the presence and absence of the selection antibiotic. The strains were grown to logarithmic phase in Middlebrook 7H9 media containing KAN, centrifuged, washed 3X with phosphate buffered saline (PBS) and diluted to an optical density (OD_600_) of 0.05 (∼ 6^∗^10^6^ CFU/ml) in 7H9 media. The diluted culture was then added to 96-well plates at a final volume of 100 μl per well with either KAN or no KAN. White and black 96-well solid bottom plates (Corning) were used for measuring luminescence and mCherry fluorescence, respectively. The plates were incubated at 37°C and 5% CO_2_. To avoid evaporation artifacts such as edge effects, the plates were kept in a humidifying box that contained reservoirs filled with water. This incubation method was followed for other experiments throughout this manuscript. Signal was read at different time intervals (0, 24, 48, and 72 h) using a Biotek Synergy H4 plate reader. The red fluorescent signal was measured at excitation/emission wavelengths of 575/620 nm and bioluminescence was measured as relative light units (RLUs) with the reading time of 1 s. After measuring the signal, an aliquot was removed from each well (both with and without KAN selection) at the indicated times, serially diluted and plated on Middlebrook 7H10 KAN agar plates.

### Susceptibility to Organic Solvents

The *M. abscessus* 390S strain was examined for susceptibility to three commonly used organic solvents- dimethylsulfoxide (DMSO), dimethylformamide (DMF) and acetone. A logarithmic culture of 390S-*lux* was diluted to an OD of 0.01 (∼2 × 10^6^ CFU/ml) and added to a range of 0.5–10% of DMSO, DMF and acetone in a white solid bottom 384 -well plate. Controls containing no organic solvent (negative control) or AMK (positive control) were included. The plates were incubated at 37°C and luminescence was read at 0, 24, 48, and 72 h using a Biotek plate reader. Susceptibility to organic solvents was calculated as percent inhibition using the formula [(negative control signal - sample signal)/negative control signal ^∗^ 100].

### Reporter Signal Optimization Assay

The fluorescence and luminescence signal readouts were optimized for the 390S strain in a 384-well plate with different starting inoculum sizes and volumes. Logarithmic phase cultures of 390S-*lux* and 390S-mCh were diluted to three different ODs -0.01, 0.05 and 0.1 – in Middlebrook 7H9 broth. The diluted culture was then added to appropriate plates at three different volumes -30, 50, and 70 μl per well – for luminescence and mCherry fluorescent signal. The signal was read at time intervals of 0, 24, 48, and 72 h of incubation using a Biotek plate reader using the parameters described earlier. AMK (32 μg/ml) was included as a positive control. A Z-factor, a statistical parameter of HTS quality control was calculated for each time point, OD and volume as described previously (Zhang et al., 1999). A Z-factor quantifies the quality of an assay and it takes into account the assay signal dynamic range and data variation associated with the signal. A Z-factor value of 0.5 and above is indicative of a robust HTS assay.

### Reporter-Based Minimum Inhibitory Concentration (MIC) Assay

Minimum inhibitory concentrations (MIC) were determined with CLR and AMK, clinically relevant antibiotics known to be active against *M. abscessus*. A two-fold serial dilution of the antibiotic in a 16-step process was carried out for CLR (128-0.0039 μg/ml) and AMK (256-0.0078 μg/ml). *M. abscessus* 390S-*lux* and 390R-*lux* cultures were diluted from a logarithmic-phase culture in Middlebrook 7H9 broth and added to the appropriate 384–well microtiter plates at a final OD_600_ of 0.01 in a total volume of 30 μl per well. An untreated control was also included. The plates were incubated at 37°C and luminescence and fluorescence was read at 0, 24, 48, and 72 h. A modified Gompertz model was used to fit the data and to generate dose-response curves using GraphPad Prism (Lambert and Pearson, 2000). MIC, defined as the lowest drug concentration at which more than 99% of bacterial growth is inhibited, was calculated from the fitted curve as compared to the untreated control.

### Small Scale Pilot Screen and Hit Validation

A library of 2,093 natural product-like compounds obtained from Asinex were screened for activity against *M. abscessus*. *M. abscessus* 390S-*lux* culture diluted to an OD_600_ of 0.01 was added to a 384-well solid bottom white plates containing compounds at a final concentration of 34 μg/ml in 30 μl volume. The compounds and the culture were added using a liquid robotic handler (Precision XS 2000, Biotek). DMSO (2%) and amikacin controls were also included in the screening plates. The screening plates were incubated for a period of 72 h. Bioluminescence was measured after every 24 h. Percent inhibition and Z-factor were calculated as described earlier. A compound exhibiting inhibition ≥ 50% relative to the untreated control are considered potential hit candidates.

An *in vitro* killing assay was employed to confirm the hits obtained from the Asinex library screen. Six best hits that showed > 70% inhibition at all time points in the HTS bioluminescent screen were added at 34 μg/ml (100 μM) to the *M. abscessus* 390S-*lux* culture of OD_600_ 0.01 in a 384-well solid bottom white plate and incubated at 37°C for 48 h. A DMSO vehicle control was also included. Luminescence was measured and aliquots from each well were serially diluted in PBS of which 50 μl of dilutions (10^-1^–10^-8^) were plated onto Middlebrook 7H10 quad-plates. Colonies were counted after 5 days and percent inhibition relative to its respective DMSO control was calculated. MIC determination of the selected hits (34.0–0.001 μg/ml) was carried out as described earlier while maintaining the final 2% DMSO throughout the dilution.

## Results

### Characterization of Reporter Strains

**FIGURE 1:**
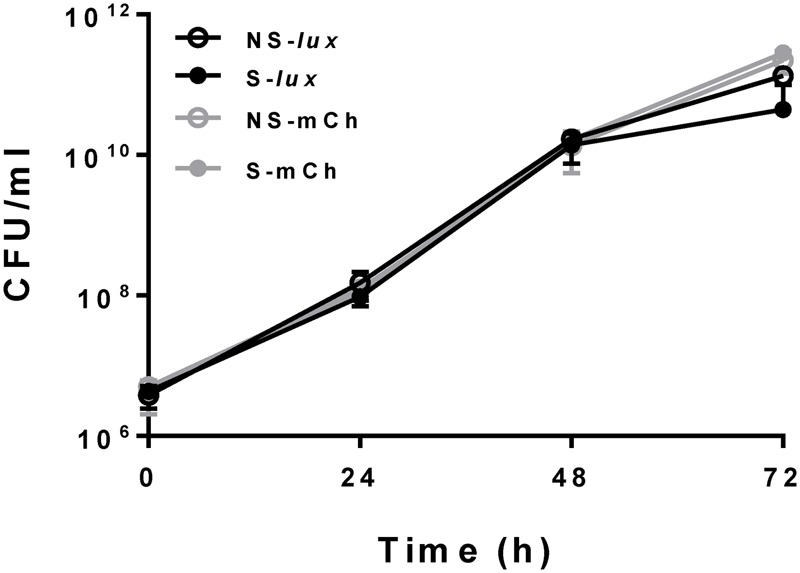
Reporter plasmids stability. Bacterial growth in CFU/ml with *M. abscessus* 390S-*Lux* (black) and 390S-mCh (gray) reporter strains with selection (S, solid circle) and no selection (NS, open circle) of the antibiotic KAN. The data is an average of three independent experiments and standard deviation is represented by error bars.

We constructed fluorescent reporter strains 390S-mCh/390R-mCh (episomal mCherry expression) and luminescent reporter strains 390S-*lux*/390R-*lux* (chromosomal expression of the entire *luxCDABE* operon). An episomal mCherry construct, rather than an integrated construct was utilized to achieve acceptable S/B ratios due to multiple plasmid copies per bacterial cell. Because we intended to avoid antibiotic selection for reporter plasmids during antimicrobial assays, we first examined the stability of the plasmids in the absence of selective pressure to ensure consistent signal output. To do this, we measured the fluorescent (mCherry) and luminescent (Lux) signal output over time with and without antibiotic selection (KAN). The steady signal ratio ∼1 under +KAN/-KAN conditions over a period of 72 h indicated little to no loss of plasmid in the absence of selection (**Table 2**). This was confirmed by plating the cultures onto KAN containing media. We observed no difference in CFU when 390S-mCh and 390S*-lux* cultures were grown with or without KAN selection for a 72 h period (**Figure 1**). This indicates that both the episomal fluorescent and integrative bioluminescent plasmids are stable in the absence of the selection antibiotic for at least 72 h and therefore, are suitable for use in testing antimicrobial activity of drugs.

**Table 2:** Signal ratio with ±KAN.

Time (h)	Lux	mCherry
0	0.96 ± 0.06	1.00 ± 0.01
24	0.97 ± 0.18	0.86 ± 0.10
48	1.01 ± 0.08	1.01 ± 0.06
72	1.08 ± 0.05	1.18 ± 0.03

### Susceptibility to Organic Solvents

We examined the tolerance of *M. abscessus* 390S reporter strains for organic solvents commonly used to dissolve compounds in most drug libraries. We exposed 390S-*lux* to DMSO, DMF, and acetone at concentrations ranging from 0 to 10% for 72 h. We found that *M. abscessus* was highly susceptible to DMF followed by DMSO and acetone (Supplementary Figure S1). Inhibition > 20% was taken as an indication of toxicity not suitable for drug screening purposes. DMF concentration above 0.5% showed inhibition greater than the 20% cut-off. In contrast, DMSO did not result in significant inhibition even when the concentration was increased to 2% which is four-fold higher than deleterious DMF concentrations. Acetone was very well-tolerated and did not result in inhibition greater than 20% even at the highest concentration of 10%. This information is critical to guide decisions regarding choice of solvents and appropriate concentrations to use in HTS drug assays without compromising the growth of *M. abscessus*.

### Optimization of 384-Well *M. abscessus* HTS Assays

**FIGURE 2:**
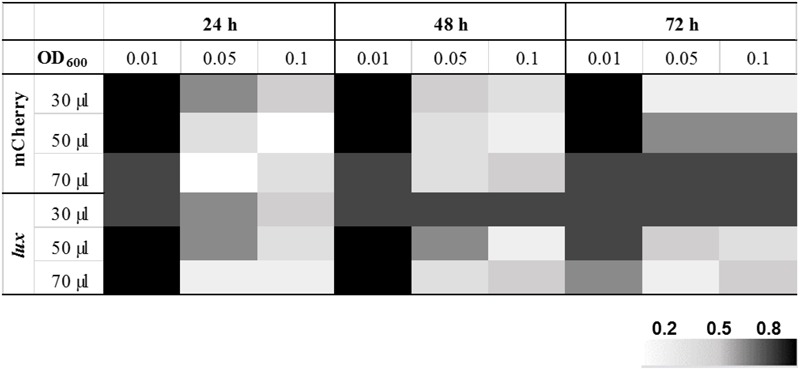
Optimization of HTS parameters. Heat map of Z-factor values from optimization of fluorescent mCherry and the bioluminescent Lux reporter screen with the *M. abscessus* 390S strain. The data is an average of three independent experiments.

**FIGURE 3:**
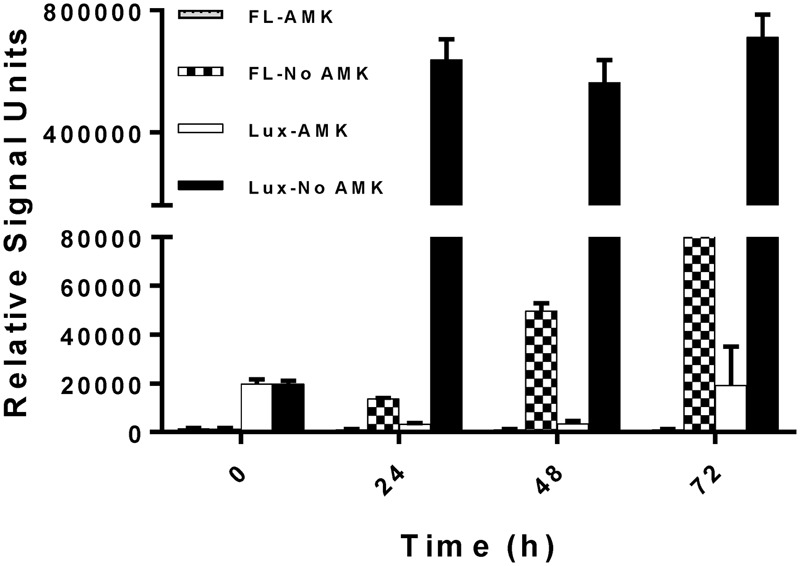
Kinetics of reporter signal output. Fluorescent (FL) and bioluminescence (Lux) signal with and without the drug AMK with *M. abscessus* 390S-mCh and 390S-*lux* strain. The data is an average of three independent experiments and standard deviation is represented by error bars.

After ensuring that the reporter plasmids are stable, we optimized the drug screening conditions in a 384-well plate for both the reporter strains (390S-*lux* and 390S-mCh). Several parameters such as the starting inoculum, volume and incubation time were simultaneously evaluated to achieve an optimal Z-factor > 0.5. Three different cell densities of 390S-mCh and 390S-*lux* (0.01, 0.05 and 0.1) were tested with different volumes (30, 50, and 70 μl) and at different times (24, 48, and 72 h). We obtained an excellent Z-factor close to 0.8 with cultures of OD_600_ 0.01 irrespective of the sample volume and incubation time (**Figure 2** and Supplementary Table S1). Z-factors dropped with an increase in OD above 0.01. We also achieved a robust gain of signal in the untreated control versus the AMK-treated control with both the reporters (**Figure 3**). The fluorescence signal reached the highest intensity after 72 h while the luminescence signal reached saturation after 24 h (**Figure 3**). We also determined the signal-to-background ratios (S/B) with each optimized parameter to assess the background contribution to the signal readout (Supplementary Table S2). A minimum signal of 10-fold over the background was achieved with both signal types for *M. abscessus* 390S strain indicating assay reliability. We observed S/B ratios with luciferase at least 70-fold higher than with fluorescent mCherry as expected. Based on the Z-factor and signal intensity, we selected the starting inoculum of OD_600_ 0.01 and 30 μl volume for both assays, with incubation times of 72 and 48 h for the fluorescence and luminescence read-out assay, respectively. The choice of the smallest working volume (30 μl) was also based on maximizing miniaturization and cost-effective use of compounds. Reproducible S/B ratios > 10 and Z-factors > 0.8 with both fluorescent and luminescent reporters indicates two robust, 384-well screening assays. The bioluminescence reporter was used for further validation studies because of sensitivity.

### MIC Determination of Known Antibiotics Using Bioluminescent Reporter

To validate our assays for drug susceptibility testing, we determined the MIC of two antibiotics, the macrolide CLR and the aminoglycoside AMK, using bioluminescent *M. abscessus* strains (390S-*lux* and 390R-*lux*). These antibiotics were chosen for two reasons. First, their MIC is well documented which allows us to validate MIC determined via the reporter-based assay. Second, they are clinically relevant front-line treatment options for *M. abscessus* infections (Brown et al., 1992; Maurer et al., 2012; Lee et al., 2015). Dose-response curves are shown in Supplementary Figure S2 and MIC values are listed in **Table 3**. The range of MIC values determined with the smooth variant for the 24–72 h time period is 0.2–2 μg/ml for CLR and 4–10 μg/ml for AMK. The MIC values of amikacin are at least 5-fold higher compared to clarithromycin with both the *M. abscessus* morphotypes consistent with the previous reports (Greendyke and Byrd, 2008; Ferro et al., 2015). An increase in MIC values was observed with the length of incubation time as noted by others with *M. abscessus*, other bacteria and fungi (Kenny and Cartwright, 1993; Pujol et al., 1997; Paphitou et al., 2002; Egervarn et al., 2007; Lee et al., 2014). We did not observe differences in the MIC values for CLR and AMK between the two strains, 390S and 390R (**Table 3**). Slightly higher MIC exhibited by 390R in contrast to 390S was found to be statistically insignificant (one-sample *t*-test).

**Table 3:** Minimum inhibitory concentration (MICs) (μg/ml) of CLR and AMK with *M. abscessus-lux* strains.

		24 h	48 h	72 h
CLR	390S	0.33	0.72	1.88
	390R	0.28	0.99	3.14
AMK	390S	4.21	7.65	9.21
	390R	8.73	8.89	13.13

### Pilot Screen of Natural Product Inspired Drug Library

**FIGURE 4:**
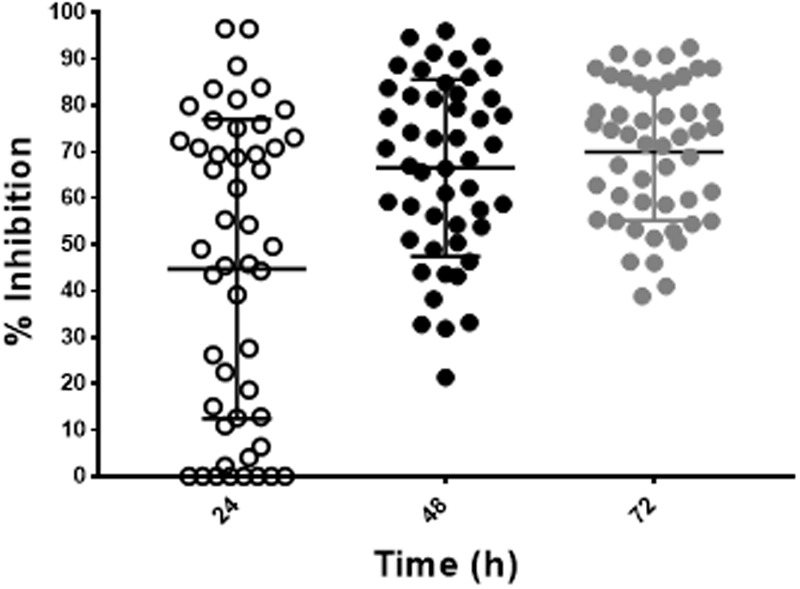
Discovery of hit compounds. A scatter plot showing percent inhibition of the hit compounds (49) identified from the Asinex library at 24, 48, and 72 h.

**FIGURE 5:**
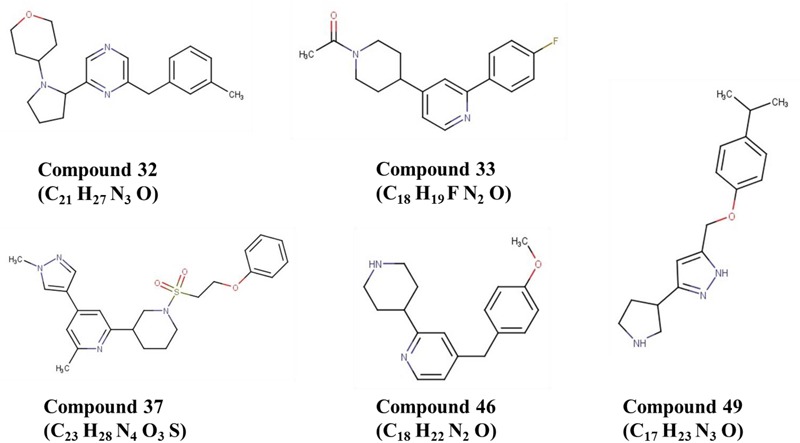
Structures of five validated hits.

To validate the utility of our assay for *M. abscessus* drug discovery, a pilot screen of 2,093 diverse natural product-inspired scaffolds (Asinex) was carried out for activity against *M. abscessus* (390S) at a concentration of 34 μg/ml. The bioluminescence signal was used to monitor bacterial growth/inhibition after every 24 h over a period of 72 h with the intent to capture potent hits early on and less potent ones at later time-points. Loss of signal as compared to the untreated control indicates an active compound. Z-factor calculated across all the drug plates were above 0.5 over the course of 72 h indicating robustness of the screen. We obtained a total of 49 hits (hit rate of 2.3%) based on the cutoff of ≥50% inhibition relative to the DMSO control (**Figure 4** and Supplementary Table S3). Five hits showed > 90% inhibition of *M. abscessus* with two appearing at 24 h and 3 hits at 48 h. Similarly, the number of hits with >80% inhibition were higher at 48 h than at 24 and 72 h (**Table 4**). We chose to validate 6 potent hits that exhibited > 70% inhibition at all time points from the primary screen by repeating the luminescence based assay and also by enumerating CFU. Five of the six selected hits showed greater than 50% percent inhibition compared to the DMSO control in both of the two assays (luminescence and CFU) after 48 h incubation (**Table 5**) indicating the validity of the reporter-based assay. The structures, molecular formulas and SMILES ID of the five validated compounds are shown in **Figure 5** and Supplementary Table S4. We also determined MIC values (**Table 5**) to better rank the hits based on potency. Hit compound **33** appeared to be most potent indicated by the lowest MIC value of 4.8 μg/ml (**Table 5**), a reasonable starting point for hit-to-lead development.

**Table 4:** Hits obtained from an Asinex library screening.

	Percent inhibition			
Time (h)	≥50	≥80	≥90	Z-factor^∗^
24	23	6	2	0.6
48	39	15	5	0.7
72	45	13	4	0.7

*^∗^Average Z factor from all 384-well plates.*

**Table 5:** Selected hits from an Asinex drug screening.

Compound name	% inhibition (Lux)	% inhibition (CFU/ml)	MIC in μg/ml (Lux)
10	83.4	23.4	8.8
32	60.3	63.5	10.4
33	85.3	56.5	4.8
37	90.6	53.2	12.0
46	92.7	72.2	10.7
49	82.9	53.4	15.3

## Discussion

The problem of inherent antibiotic resistance coupled with the lack of efforts to identify new compounds active against the emerging opportunistic pathogen *M. abscessus* presents a big challenge. This urgent need for effective therapeutic options for *M. abscessus* infections requires robust tools for drug discovery. In pursuit of this goal, we developed two reporter-based high-throughput screening assays for simple, reliable and faster screening of drug libraries for activity against *M. abscessus*.

Fluorescent and bioluminescent reporters have been used extensively to test antimicrobial activity of compounds against several mycobacteria and other related bacteria but surprisingly not for *M. abscessus* (Cooksey et al., 1995; Arain et al., 1996; Shawar et al., 1997). In this study, we demonstrated the feasibility of using fluorescent mCherry and luciferase reporters for HTS to identify novel antimicrobials against *M. abscessus*. Comparison of AMK treated versus untreated control revealed a high signal/background ratio (S/B) for both the readouts (Supplementary Table S2), supporting the use of either reporter in *M. abscessus* HTS assays. The *lux* signal was more sensitive than mCherry as indicated by raw signal strength and S/B ratios (at least 70-fold higher than mCherry) (**Figure 3** and Supplementary Table S2). However, unlike mCherry the Lux signal seemed to plateau after 24 h (**Figure 3**) even though there was an increase in cell numbers evident by increased CFU with time (**Figure 1**). There is a dynamic range for luminescence signal detection of 6 orders of magnitude based on the instrument limitations and auto-scaling of gain settings. The plateau observed for the *lux* signal was likely due to saturation of the plate reader detectors. Nevertheless, the study demonstrated the use of both mCherry and Lux signal as suitable readouts for *M. abscessus* drug screening based on parameter such as Z-factor, signal strength and S/B.

Because HTS assays are almost never done in duplicates, a high Z-factor provides assurance of finding hits with confidence from a very large number of compounds. In the present study, we rigorously optimized assay parameters to achieve an excellent Z-factor of 0.8 (Supplementary Table S1). It is worth noting that this Z-factor was achieved with a minimum volume (30 μl). This is important from the miniaturization and cost-effectiveness standpoint of a HTS assay. This study showed that starting inoculum density greatly influenced Z-factor with higher inoculum > OD_600_ 0.01 resulting in values lower than 0.8 irrespective of the sample volume-per-well and the incubation time. It is likely that cells clump with increased cell density under static incubation conditions and thereby introduce more variation in signal measurement. Consistent with this idea, we experienced lower Z-factors (below 0.5) with the *M. abscessus* 390R, a strain known to form clumps. An inclusion of a de-clumping step improved the Z-factor values.

Determination of MIC values is an important aspect of hit characterization to assess drug efficacy and potential therapeutic window of a compound. In this study, we demonstrated that MIC determined with bioluminescent reporter agrees well with MIC determined through non-reporter based methods (**Table 3**) (Choi et al., 2012; Maurer et al., 2014; Lefebvre et al., 2016). For instance, the MIC value of 7.6 μg/ml for AMK at 48 h is almost identical to the previously reported MIC of 8 μg/ml for the same incubation time (Lefebvre et al., 2016). Similarly, the MIC of CLR (1.88 μg/ml) is in accordance with the published MIC value of 2 μg/ml after 72 h incubation (Choi et al., 2012). MIC assessment from 16-point dilutions of drug compounds in a 384-well format carried out in this study can be very well semi-automated using a robotic liquid handler. This will enable faster validation and prioritization of potent hits among a pool of hits obtained from a library screen. MIC determination with less expenditure of resources and efforts can be instrumental in hit discovery to hit-to-lead process. In this study, we also assessed strain-specific effects on MIC values by employing the invasive non-biofilm former, *M. abscessus* 390R and the biofilm forming 390S strain. We did not observe a significant difference in MIC values between the rough (390R) and smooth (390S) strains of *M. abscessus* which is consistent with the previous reports investigating multiple drugs like AMK, CLR, cefoxitin and capuramycin (Dubuisson et al., 2010; Ruger et al., 2014). Therefore, we employed the smooth variant (390S) which clumps less and is easy to work with in our pilot screening of natural-product inspired compounds. Besides MIC determination, differentiation between bacteriostatic and bactericidal nature of compounds using reporters is feasible and has been reported previously using bioluminescent strains of *M. tuberculosis* (Andreu et al., 2012).

Although both assays (mCherry and luciferase) were suitable for HTS, we elected to use the bioluminescent assay to conduct a pilot screen due to its superior dynamic range and sensitivity. In addition, it minimizes the potential for interference from autofluorescent or fluorescence-quenching compounds (Choy et al., 2003; Simeonov et al., 2008). The screen of 2,093 natural product inspired scaffolds identified 49 hits with >50% inhibition (**Figure 4** and Supplementary Table S3), out of which 6 of the most potent hits were chosen for validation. Five (compounds **32, 33, 37, 46** and **49**) of the 6 selected hits remained valid hits with inhibition above 50% threshold in the CFU assay (**Table 5**). Failure to validate compound **10** by the CFU assay despite apparent *M. abscessus* growth inhibition by luminescence readout could result from loss of activity of labile compound during storage or due to bacteriostatic effect that would dampen luminescence increase but not decrease viable CFU counts. The 5 validated hits do not appear to have rapid bactericidal activity as evident by less than 90% inhibition by CFU after 48 h treatment time. This is not surprising given the highly drug resistant nature of *M. abscessus*. This could also be due to the short duration of both luminescence and CFU assays. Chemical database (ChemSpider) and literature searches (PubMed) did not reveal any antimicrobial activity reported for these 5 compounds. In future, we will procure related analogs of these validated hits from the Asinex compound collections and assess activity against *M. abscessus* and *M. tuberculosis*.

Overall, we carried out extensive optimization to standardize the high-throughput drug screening platform for *M. abscessus* in a 384-well format. This HTS can be reliably used as a primary screen to rapidly and effectively screen large libraries for compounds with biological activity against *M. abscessus*. To our knowledge this is the first reporter-based whole-cell drug screening study against this notorious pathogen. We are already using these assays to conduct drug discovery projects of large natural product libraries, with promising hits from early results. This drug-discovery approach is likely to pave a way for identification and development of new antimicrobials.

## Author Contributions

All authors contributed to the design of the study, analysis and/or interpretation of the data; TB and KR provided critical resources and reagents; RG and MN were responsible for conducting the experiments and data acquisition; All authors participated in the writing, critically reviewing, and editing of the manuscript.

## Conflict of Interest Statement

The authors declare that the research was conducted in the absence of any commercial or financial relationships that could be construed as a potential conflict of interest.

## Footnotes

1 www.asinex.com

2 www.molsoft.com/icm_pro.html
