# Supplementary material for: Reporter-Based Assays for High-Throughput Drug Screening against Mycobacterium abscessus
Source: Front Microbiol. 2017 Nov 10;8:2204. doi: 10.3389/fmicb.2017.02204 (PMC5687050; doi:10.3389/fmicb.2017.02204)
Supplement: Supplementary file 1 [file Table_1.PDF]

Table S1: Z-factor based optimization of *Mab* HTS assays

		24 h			48 h			72 h		
	**OD_{600}**	**0.01**	**0.05**	**0.1**	**0.01**	**0.05**	**0.1**	**0.01**	**0.05**	**0.1**
	**390S**									
	30 µl	0.8	0.6	0.5	0.8	0.5	0.4	0.8	0.3	0.3
**mCherry**	50 µl	0.8	0.4	0.2	0.8	0.4	0.3	0.8	0.6	0.6
	70 µl	0.7	0.2	0.4	0.7	0.4	0.5	0.7	0.7	0.7
	30 µl	0.7	0.6	0.5	0.7	0.7	0.7	0.7	0.7	0.7
lux	50 µl	0.8	0.6	0.4	0.8	0.6	0.3	0.7	0.5	0.4
	70 µl	0.8	0.3	0.3	0.8	0.5	0.5	0.6	0.3	0.5
	**390R**									
mCherry	30 µl	0.7	nd	nd	0.7	nd	nd	0.5	nd	nd
lux	30 µl	0.8	nd	nd	0.8	nd	nd	0.7	nd	nd

*nd means not determined
