# Supplementary material for: Reporter-Based Assays for High-Throughput Drug Screening against Mycobacterium abscessus
Source: Front Microbiol. 2017 Nov 10;8:2204. doi: 10.3389/fmicb.2017.02204 (PMC5687050; doi:10.3389/fmicb.2017.02204)
Supplement: Supplementary file 2 [file Table_2.PDF]

**Table S2: Signal-to-background ratio in HTS**

| | | 24 h | | | 48 h | | | 72 h | | |
|---|---|---|---|---|---|---|---|---|---|---|
| | **$OD_{600}$** | **0.01** | **0.05** | **0.1** | **0.01** | **0.05** | **0.1** | **0.01** | **0.05** | **0.1** |
| | **390 S** | | | | | | | | | |
| | 30 µl | 12 | 16 | 19 | 39 | 51 | 62 | 106 | 91 | 101 |
| ***mCherry*** | 50 µl | 10 | 16 | 27 | 34 | 43 | 50 | 84 | 95 | 93 |
| | 70 µl | 10 | 21 | 34 | 37 | 48 | 59 | 78 | 104 | 111 |
| | 30 µl | 4046 | 5034 | 5607 | 7298 | 10648 | 12199 | 8167 | 9415 | 9535 |
| ***lux*** | 50 µl | 2418 | 2973 | 4101 | 6345 | 6804 | 7208 | 6211 | 6881 | 6763 |
| | 70 µl | 1897 | 2766 | 5095 | 6607 | 7660 | 9021 | 7799 | 7379 | 7539 |
| | **390 R** | | | | | | | | | |
| ***mCherry*** | 30 µl | 12 | nd | nd | 7 | nd | nd | 6 | nd | nd |
| ***lux*** | 30 µl | 1136 | nd | nd | 2866 | nd | nd | 6214 | nd | nd |

*nd means not determined
