# Supplementary material for: Reporter-Based Assays for High-Throughput Drug Screening against Mycobacterium abscessus
Source: Front Microbiol. 2017 Nov 10;8:2204. doi: 10.3389/fmicb.2017.02204 (PMC5687050; doi:10.3389/fmicb.2017.02204)
Supplement: Supplementary file 3 [file Table_3.PDF]

Table S3: Hits identified from an Asinex drug library screening

	Percent Inhibition		
**Compoun**	**24 h**	**48 h**	**72 h**
1	49.0	77.9	85.1
2	22.5	54.3	74.4
3	70.9	66.4	73.8
4	66.2	77.5	86.5
5	69.4	73.0	78.5
6	45.8	82.5	71.2
7	70.9	74.2	78.4
8	88.6	86.1	86.4
9	62.2	81.4	75.3
**10**	81.3	96.1	91.1
11	83.6	83.8	90.3
12	69.3	87.7	88.0
13	76.0	67.0	76.0
14	66.2	90.0	88.1
15	43.5	68.3	64.1
16	79.2	77.1	85.9
17	0.0	46.3	60.5
18	18.7	65.6	66.7
19	2.2	53.8	53.2
20	4.1	50.4	55.0
21	55.4	71.6	77.9
22	44.3	44.1	52.7
23	0.0	21.3	68.9
24	0.0	43.2	67.1
25	6.4	33.2	59.7
26	0.0	38.2	54.4
27	10.9	43.6	55.1
28	0.0	81.5	84.7
29	0.0	32.7	55.4
30	0.0	31.9	59.2
31	15.0	51.0	51.3
**32**	96.5	70.7	84.0
**33**	83.8	94.7	78.7
34	49.6	56.2	58.6
35	73.1	88.7	88.1
36	0.0	57.5	71.7
**37**	96.6	91.3	74.7
38	72.4	79.3	73.2
39	54.3	58.7	61.4
40	12.6	59.2	38.9
41	27.6	62.2	46.0
42	26.2	58.3	41.0
43	76.8	84.8	76.7
44	68.8	82.1	77.7
45	12.8	61.0	46.3
**46**	75.1	88.2	90.7
47	45.4	72.9	62.8
48	39.2	48.9	50.6
**49**	79.9	92.7	92.5

Bold respresents the selected hits for confirmation
