# Supplementary material for: Reporter-Based Assays for High-Throughput Drug Screening against Mycobacterium abscessus
Source: Front Microbiol. 2017 Nov 10;8:2204. doi: 10.3389/fmicb.2017.02204 (PMC5687050; doi:10.3389/fmicb.2017.02204)
Supplement: Supplementary file 4 [file Table_4.PDF]

Table S4: Selected hits from an Asinex drug screening

Compound Name	SMILES
32	CC1=CC(CC2=CN=CC(=N2)C2CCCN2C2CCOCC2)=CC=C1
33	CC(=O)N1CCC(CC1)C1=CC=NC(=C1)C1=CC=C(F)C=C1
37	CN1C=C(C=N1)C1=CC(=NC(C)=C1)C1CCCN(C1)S(=O)(=O)CCOC1=CC=CC=C1
46	COC1=CC=C(CC2=CC(=NC=C2)C2CCNCC2)C=C1
49	CC(C)C1=CC=C(OCC2=CC(=NN2)C2CCNC2)C=C1

* details can be accesed by SMILES using Chemspider.com
