# Supplementary material for: Reporter-Based Assays for High-Throughput Drug Screening against Mycobacterium abscessus
Source: Front Microbiol. 2017 Nov 10;8:2204. doi: 10.3389/fmicb.2017.02204 (PMC5687050; doi:10.3389/fmicb.2017.02204)
Supplement: Supplementary file 5 [file Image_1.PDF]

Figure S1

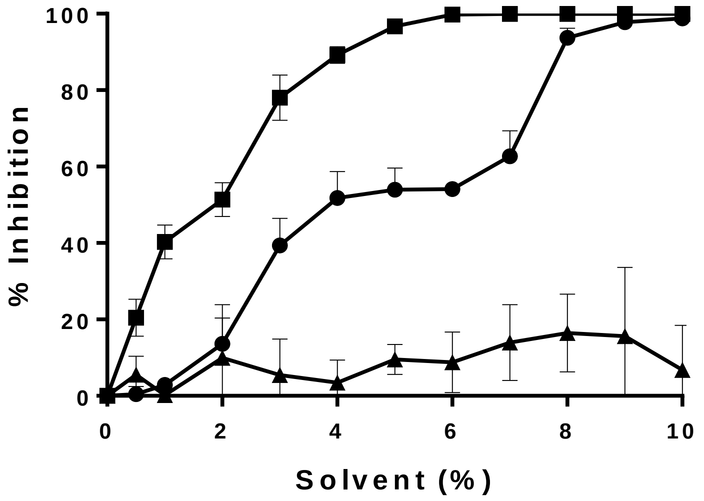

Fig. S1: Organic solvents tolerability. Percent inhibition relative to the untreated control with the *Mab* 390S-*lux* strain when exposed to 0-10 % concentrations of DMF (square), DMSO (circle), and acetone (triangle) for 72 h. The data is an average of three independent experiments and standard deviation is represented by error bars.
