# Supplementary material for: Reporter-Based Assays for High-Throughput Drug Screening against Mycobacterium abscessus
Source: Front Microbiol. 2017 Nov 10;8:2204. doi: 10.3389/fmicb.2017.02204 (PMC5687050; doi:10.3389/fmicb.2017.02204)
Supplement: Supplementary file 6 [file Image_2.PDF]

Figure S2

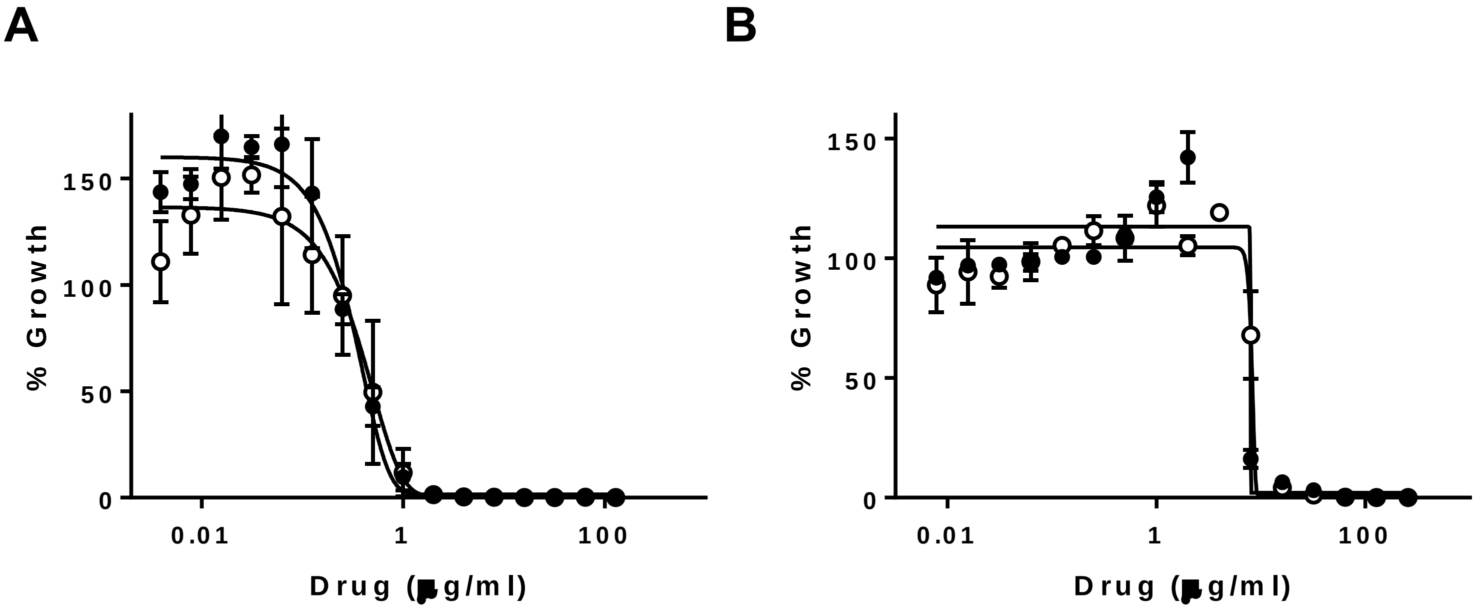

Fig. S2: Dose-response curves of antibiotics CLR and AMK. *Mab* strains, 390S-*lux* (closed circle) and 390R-*lux* (open circle), were exposed to the drugs CLR **(A)** and AMK **(B)** for 72 h and growth was measured as RLU. Percent growth was calculated as compared to the untreated control. These curves were used to determine MIC by Graph Pad Prism. The data is an average of three independent experiments and standard deviation is represented by error bars.
